# Conserving avian evolutionary history can effectively safeguard future benefits for people

**DOI:** 10.1126/sciadv.adh4686

**Published:** 2023-09-20

**Authors:** Rikki Gumbs, Claudia L. Gray, Michael Hoffmann, Rafael Molina-Venegas, Nisha R. Owen, Laura J. Pollock

**Affiliations:** ^1^ Conservation and Policy, Zoological Society of London, London NW1 4RY, UK.; ^2^ Science and Solutions for a Changing Planet DTP, Grantham Institute, Imperial College London, London SW7 2AZ, UK.; ^3^ IUCN SSC Phylogenetic Diversity Task Force, London, UK.; ^4^ Department of Ecology, Faculty of Science, Universidad Autónoma de Madrid, Madrid, Spain.; ^5^ On the Edge Conservation, London SW3 2JJ, UK.; ^6^ Department of Biology, McGill University, Montréal, Québec H3A 1B1, Canada.

## Abstract

Phylogenetic diversity (PD)—the evolutionary history of a set of species—is conceptually linked to the maintenance of yet-to-be-discovered benefits from biodiversity or “option value.” We used global phylogenetic and utilization data for birds to test the PD option value link, under the assumption that the performance of sets of PD-maximizing species at capturing known benefits is analogous to selecting the same species at a point in human history before these benefits were realized. PD performed better than random at capturing utilized bird species across 60% of tests, with performance linked to the phylogenetic dispersion and prevalence of each utilization category. Prioritizing threatened species for conservation by the PD they encapsulate performs comparably to prioritizing by their functional distinctiveness. However, species selected by each metric show low overlap, indicating that we should conserve both components of biodiversity to effectively conserve a variety of uses. Our findings provide empirical support for the link between evolutionary history and benefits for future generations.

## Teaser

Empirical evidence that conserving avian evolutionary history can effectively maintain future options.

## INTRODUCTION

Biodiversity contributes a wide variety of benefits and services to humanity including food, fuel, medicine, materials, and a myriad other economic and cultural values (*1*, *2*). Unfortunately, humanity’s reliance on biodiversity is now a major driver of the unprecedented declines across species and ecosystems globally (*3*, *4*). Accordingly, the goal of maintaining the benefits contributed by biodiversity for current and future generations, through conservation and sustainable use, now sits at the heart of global biodiversity policy (*5*), including as part of the recently adopted Kunming-Montreal Global Biodiversity Framework under the Convention on Biological Diversity (*6*).

There are many ways to value biodiversity and nature in general (*7*), the most prominent of which is through ecosystem services (*8*). However, the values and benefits of species are difficult to assess, particularly for those benefits that are indirect or emerge unexpectedly (*1*), such as increased coffee yield through the maintenance of insectivorous bird populations to act as pest control (*9*). These unexpected benefits highlight the importance of maintaining the option to use benefits in the future that are currently unknown or unexploited, that is, the “option value” of biodiversity (*8*), and explicitly link the maintenance of biodiversity now to the capacity for future generations to benefit from them (*10*, *11*).

Given that the precise nature of future options are, by their very definition, unknown and unfeasible to quantify presently, a suitable proxy available now would prove to be a valuable tool to monitor biodiversity’s capacity to provide benefits into the future. Phylogenetic diversity (PD)—which theoretically approximates the suite of features shared by, and unique to, a set of species by measuring the phylogenetic branches that connect them (*12*)—has been proposed to fulfill this role, under the assumption that maintaining a greater amount of PD will conserve distinct features and consequently a wider variety of potential benefits (*5*).

Although it is not possible to predict the precise nature of future benefits it is reasonable to assert that known benefits today were, at some point in the human history, unknown future options for humanity. For example, most biodiversity benefits today could be seen as option value for the future generations of the first humans that appeared in Africa roughly 200,000 years ago. Thus, work has been done to assess the performance of PD at capturing known benefits from plants when applied naively (i.e., selecting species for conservation based on PD with no knowledge of the distribution of benefits). Forest *et al.* (*13*) found that selecting sets of plant genera to maximize PD in the Cape of South Africa would more efficiently retain known benefits than selecting based on random sampling. In addition, Molina-Venegas *et al.* (*14*) showed that maximizing PD more efficiently safeguarded a larger number and wider variety of known benefits provided by the plant genera globally.

However, whether the predictions of the PD/biodiversity benefit framework will hold for other taxonomic groups beyond plants remains unknown. Previous research into the link between PD and the utilization of species has focused on maximizing diversity irrespective of extinction risk, largely due to the lack of extinction risk data available for the world’s plants (*15*). Nonetheless, understanding the distribution of utilized and threatened species across the Tree of Life also has the potential to guide conservation efforts to safeguard the most irreplaceable species, whose value to people may also be a major driver of their imperilment (*16*–*18*).

Here, we categorize the most comprehensive species-level dataset available of recorded consumptive uses of bird species (*19*, *20*). We split the dataset into four distinct utilization types, against which 94% of utilized species could be categorized: food and feed (hereafter “food”); materials; medicinal, biochemical, and genetic resources (hereafter “medicine”); and pets and display animals (hereafter “pets”; see Materials and Methods). We first use this dataset to determine whether selecting sets of species to maximize PD without knowledge of their utilization value can efficiently capture species with known uses and thus indicate PD’s utility for effectively maintaining both present and currently unknown options. This is a test of the hypothesis that if we, in the past, only retained sets of bird species that maximized PD to the current day, we would capture more species with benefits than if we selected sets at random. Then, we explore how conservation strategies to maximize or retain threatened PD, using contemporary extinction risk information, perform at safeguarding currently utilized and threatened bird species. This is a test of the hypothesis that prioritizing threatened bird species by PD can capture more species with currently known uses than prioritizations based on extinction risk alone, for the benefit of future populations. Last, we investigate the relative performance of strategies that target evolutionary or functionally unique species at capturing threatened and utilized bird species and any redundancy between the two approaches.

## RESULTS

### Avian evolutionary history and utilized species

There were 4331 bird species (39.2% of all bird species) with at least one type of utilization recorded on the International Union for Conservation of Nature (IUCN) Red List, 608 (5.5%) of which were threatened with extinction [vulnerable (VU), endangered (EN), and critically endangered (CR) on the IUCN Red List], albeit not necessarily by their utilization. Across our four broad utilization categories, the use of species as pets (including display animals; see Materials and Methods) was the largest category, with 3855 species recorded as used for pets (441 threatened spp.), followed by food (consumption of birds and/or their eggs; 1402 spp., 346 threatened), materials (including handicrafts, jewelry, and ornaments; 112 spp., 37 threatened), and medicinal uses (57 spp., 15 threatened).

The order Passeriformes (perching birds), which represents 60% of all bird species, comprised 36.5% of all utilization records in this study and was the most common order used for food (291 spp.; 20.8% of species used for food), materials (26 spp.; 23% of species used), and pets (1722 spp.; 45% of species used; fig. S1). Strigiformes (owls) was the most common order used for medicine (11 spp.; 19.3%). The taxonomic distribution of species used for pets had both the largest proportion represented by a single order and the largest disparity between the first- and second-richest order, with second-placed Psittaciformes (parrots) comprising only 8% of all pet utilization records (fig. S1). In comparison, no other utilization category has more than 23% of species records confined to a single order, nor as steep a decline between the richest order and all others as observed in pets (fig. S1). The order Bucerotiformes (hornbills and hoopoes) is the only order to be significantly overrepresented in each of the four utilization categories, whereas Passeriformes is the only order to be significantly underrepresented in each category (fig. S1).

Species associated with each utilization category captured less PD than random across the avian phylogeny, indicating phylogenetic clustering of utilization, with species used for pets exhibiting the greatest phylogenetic clumping and those used for materials and medicine being the least clumped (fig. S2). However, as reported for plants (*14*), utilization categories with higher phylogenetic dispersion (i.e., less clumped on the phylogeny), such as medicine and materials, exhibit increased relative gains in utilized species (fig. S2).

**Fig. 1.**
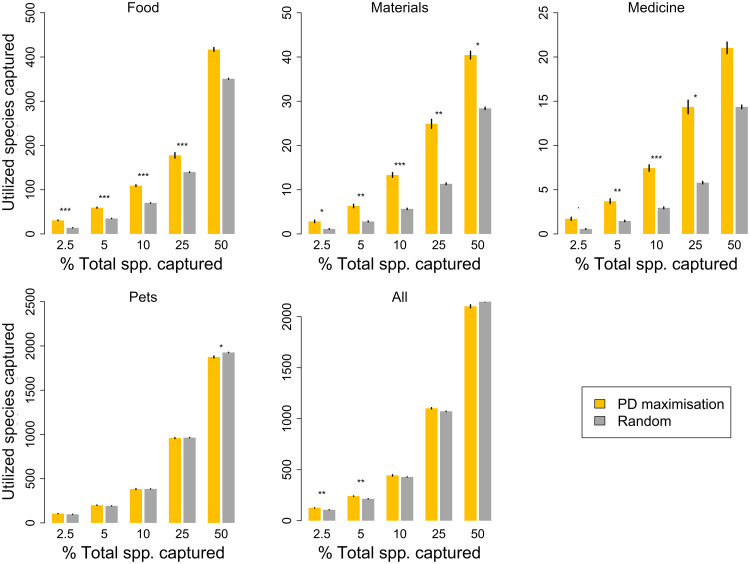
The performance of a phylogenetic diversity (PD) maximization strategy at capturing utilized bird species. The number of utilized species captured by selecting sets of species to maximize PD compared with random sets of species of the same number. Utilized species grouped into broad categories based on affinities (see Materials and Methods for details). Significance based on standardized effect size (SES) scores, for alpha of 5% (*), 1% (**), and 0.01% (***). Error bars represent 95% confidence interval around the mean for 1000 calculations.

We found that selecting sets of species to maximize PD is generally an efficient strategy for capturing utilized species of birds across the avian Tree of Life (Fig. 1), particularly at smaller sample sizes and for phylogenetically dispersed uses. At the 5% sample size, significantly more species used for food, materials, medicine—and all species from all four major utilization categories combined—are captured by maximizing PD than when selecting at random (Fig. 1). PD maximization strategies perform significantly better than random for five of five sample sizes for species used for materials, four of five for species used for food, three of five for species used for medicine. PD-maximizing strategies did not perform better than random for species used for pets, which have the highest phylogenetic clumping. Overall, PD-maximizing strategies performed significantly better than random for 12 of 20 (60%) combinations of utilization categories and sample size (Fig. 1). Including utilized species whose recorded uses could not be definitively reconciled with a distinct utilization category did not affect the results of PD maximization or threatened PD maximization for utilization records overall (fig. S3).

When comparing the evolutionary distinctiveness (ED) scores [which partitions total PD among species based on their evolutionary isolation (*21*, *22*); see Materials and Methods] of species recorded for each utilization category, species used as pets had significantly lower ED scores than those used for any other category (Kruskal-Wallis test with Dunn’s test for pairwise comparisons; adjusted *P* < 0.05 for each pairwise comparison; table S1). Species used for materials had significantly higher ED scores than those used for food, and there were no significant differences in ED between all other comparisons (table S1).

### Prioritizing threatened avian evolutionary history to safeguard utilized species

**Fig. 2.**
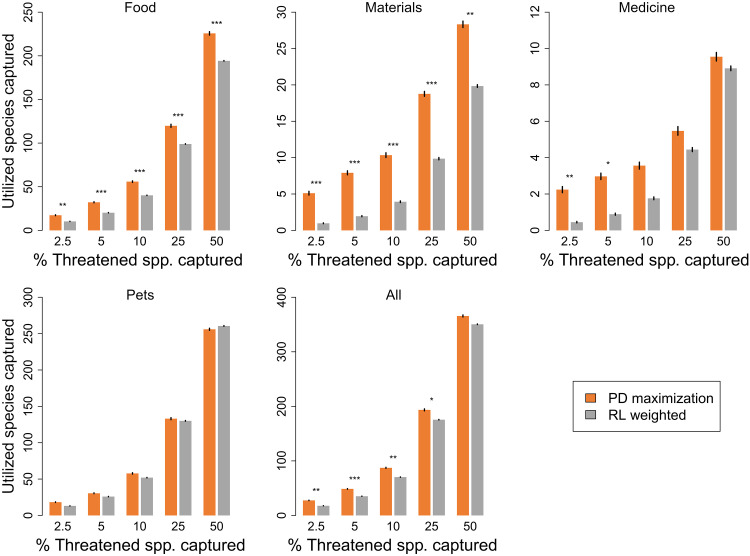
The performance of a phylogenetic diversity (PD) maximization strategy at capturing utilized and threatened bird species. The number of threatened utilized species captured by maximizing the conservation of threatened PD compared with the numbers saved when species are selected on the basis of their extinction risk (i.e., IUCN Red List category, RL weighted), with greater weighting given to species in more at risk categories (see Materials and Methods). Significance based on SES scores, for alpha of 5% (*), 1% (**), and 0.01% (***). Error bars represent 95% confidence interval around the mean for 100 values.

Given that conservation prioritization efforts typically focus on threatened species, we explored whether selecting sets of threatened species that would maximize the conservation of threatened avian PD would also efficiently capture threatened utilized species. When compared against sets of species selected using an “extinction risk-weighted” strategy (based on IUCN Red List categories, where species in higher categories of risk were more likely to be selected; see Materials and Methods), the PD maximization strategy performed significantly better across 12 of 20 combinations of utilization categories and sample (Fig. 2) and performed particularly well when smaller sets of species were selected for conservation (i.e., smaller sample sizes; Fig. 2).

The PD maximization strategy outperformed the extinction risk-weighted strategy for all sample sizes for the food and materials categories and for four of five of sample sizes for all utilized species combined (Fig. 2). However, as observed when maximizing PD for all bird species, PD maximization for threatened bird species did not outperform the extinction risk-weighted strategy for species used for pets (Fig. 2). When compared with an “extinction risk-controlled” strategy (where we selected random sets of threatened species with corresponding frequencies of Red List categories to those selected for the PD maximization set; see Materials and Methods), the PD maximization strategy performed similarly well, outperforming the extinction risk-controlled strategy in 12 of 20 of combinations (fig. S4).

**Fig. 3.**
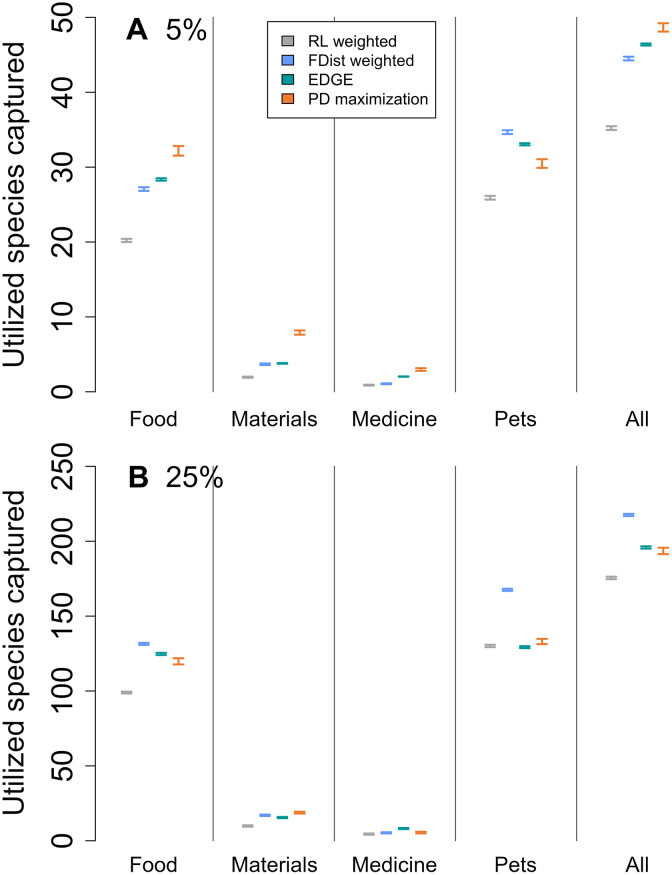
Performance of the different conservation strategies at capturing utilized and threatened species at 5 and 25% sample sizes. The number of threatened species with recorded uses for the four main utilization categories, and all groups combined, captured by each conservation strategy for the (**A**) 5% (75 spp.) and (**B**) 25% (375 spp.) sample sizes. Bars represent the 95% confidence interval from 100 iterations. Bars in order, from left to right, for each category: Extinction risk-weighted (RL weighted; gray); functional distinctiveness–weighted strategy (FDist; blue); evolutionarily distinct and globally endangered (EDGE) strategy (green); phylogenetic diversity (PD) maximization strategy (orange).

We then selected sets of species to conserve using the evolutionarily distinct and globally endangered (EDGE) metric, which incorporates extinction risk to rank species based on their ED and “global endangerment” (GE; see Materials and Methods). The EDGE approach significantly outperformed the extinction risk-weighted strategy in four of five combinations for food, two of five for materials, one of five for medicine, and zero of five for pets (7 of 20 combinations of utilization category and sample size overall; fig. S5). When compared with an extinction risk-controlled strategy, EDGE captured significantly more species in five of five combinations for food, two of five for materials, two of five for medicine, and one of five for pets (9 of 20 combinations overall; fig. S6). However, the EDGE approach did significantly outperform both extinction risk-based strategies for all utilization categories combined at all sample sizes (Fig. 3 and figs. S5 and S6).

As the targeted utilization of species can be linked to their functional uniqueness (*23*–*25*), we also tested a conservation strategy where threatened bird species were selected on the basis of their functional distinctiveness (hereafter “FDist-weighted strategy”; see Materials and Methods) for comparison with the PD maximization strategy. While the PD maximization strategy outperformed the FDist-weighted approach at small sample sizes for threatened species used for materials and medicine (Fig. 3A and fig. S7), prioritizing species by their FDist performed comparably to the PD maximization strategy and outperformed the strategy at larger sample sizes for the more phylogenetically clumped utilization categories of pets and food (Fig. 3B and fig. S7).

### Conserving distinctive and utilized species

Last, we explored the relationship between two measures of species distinctiveness, ED and FDist, and their relative performance at capturing utilized and threatened bird species. ED and FDist are significantly but weakly correlated for all birds (*r* = 0.11, df = 9644, *P* < 0.0001) and threatened birds only (*r* = 0.1, df = 1291, *P* < 0.0001). The ED and FDist of utilized species were significantly greater than that of non-utilized species (all test results for pairwise comparisons in table S2). As observed for plants (*16*), ED was significantly higher, compared with species with no recorded uses, for species used for food, materials, and medicine and for all utilization categories combined, when considering all bird species and threatened species only (table S2). Similarly, FDist is significantly higher in utilized species across all utilization categories and all categories combined when considering all bird species, but not significantly different for threatened birds used for medicine (all test results in table S3).

**Fig. 4.**
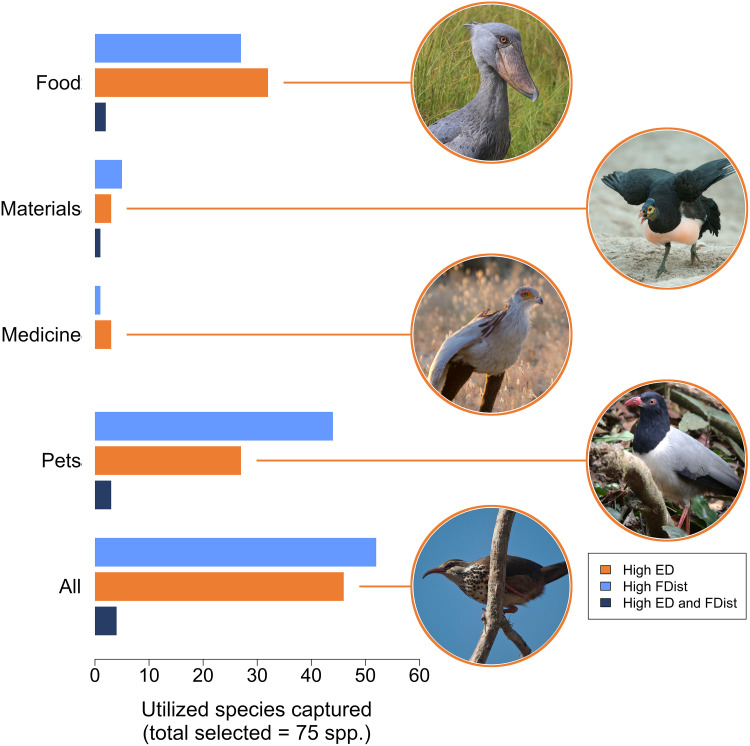
The performance of evolutionary and functional distinctiveness (FDist) at capturing utilized threatened bird species. The number of utilized species for each utilization category, and all combined, captured when 5% (75 spp.) of threatened species are selected on the basis of their evolutionary distinctivness (ED) (orange) or FDist (light blue) scores and the number of species captured by both ED and FDist (dark blue). Species featured are examples of high ED species not captured by the FDist prioritization at the 5% sample size, from top to bottom: Shoebill [*B. rex*, hunted for food (*64*)], maleo [*M. maleo*, eggs used in traditional ceremonies and as ornaments (*34*, *65*)], secretarybird [*S. serpentarius*, reports of international trade for medicinal use (*66*)]; coral-billed ground cuckoo [*Carpococcyx renauldi*, collected for the cagebird trade (*67*)]; and subdesert mesite [*M. benschi*, localized use of species for food (*68*)]. For results for all sample sizes, see fig. S8. Image credits: Shoebill, Claudia L. Gray; maleo, Kevin Schafer; secretarybird: Anthony Lowney; coral-billed ground cuckoo: Lenny Worthington; subdesert mesite: Louise Gardner.

When we selected threatened species for conservation based on their ED and FDist ranks, the two measures of distinctiveness exhibited low overlap in terms of the utilized species they prioritized, with typically <50% overlap for all utilization categories at the 2.5 to 25% sample sizes (5% sample size: Fig. 4; all sample sizes: fig. S8). If we were to prioritize 5% of threatened bird species (74 spp.) for conservation on FDist alone, we would omit 20 threatened monotypic genera (several of which are highlighted for their uses, and some of which are unexpected, in Fig. 4). Conversely, prioritizing 5% of threatened species by ED alone would omit 48 of the world’s most functionally distinct species (9% overlap of top 5% ED and FDist spp.; Fig. 4).

**Fig. 5.**
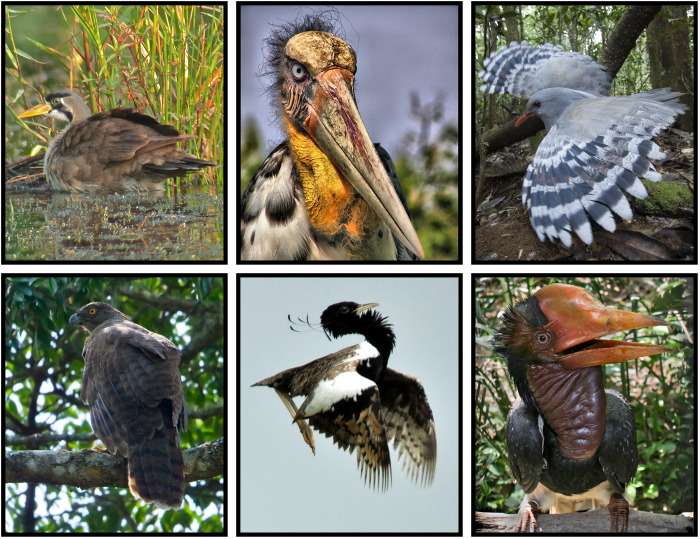
Threatened and utilized bird species in the top 2.5 to 10% of evolutionary and functional distinctiveness (FDist). A selection of utilized and threatened bird species that occur in the top 2.5% (top left: masked finfoot, *H. personatus*); 5% (top middle: greater adjutant, *L. dubius*); and 10% (top right: kagu, *R. jubatus*; bottom left: Madagascar serpent eagle, *E. astur*; bottom middle: lesser florican, *S. indicus*; bottom right: helmeted hornbill, *Rhinoplax vigil*) of evolutionary distinctiveness (ED) and FDist of threatened birds (all scores in Data S1). Image credits: Masked finfoot: Tun Pin Ong; greater adjutant: Benjamin Fitzgerald; kagu: Roger Le Guen; Madagascar serpent eagle: Dubi Shapiro; lesser florican: Parashura M. Laad; helmeted hornbill: Doug Janson.

Of the 608 threatened and utilized species included in this study, 437 (72%) also have direct exploitation listed as a threat on the IUCN Red List (*19*). One of these species, the masked finfoot (*Heliopais personatus*), is the only species in the top 2.5% of ED and FDist of utilized and threatened birds (Fig. 5). The three species in the top 5% of both distinctiveness measures, alongside the masked finfoot, are the Abyssinian ground hornbill (*Bucorvus abyssinicus*), greater adjutant (*Leptoptilos dubius*), and malleefowl (*Leipoa ocellata*). The 17 utilized and threatened species in the top 10% for both distinctiveness measures include the most evolutionarily distinct threatened bird, the kagu (*Rhynochetos jubatus*), Madagascar serpent eagle (*Eutriorchis astur*), lesser florican (*Sypheotides indicus*), and takahē (*Porphyrio hochstetteri*; Fig. 5 and data S1).

## DISCUSSION

Our findings support earlier work on plants (*13*, *14*) and suggest that maximizing PD provides a useful strategy for maintaining benefits across the world’s birds, although PD’s performance varies depending on the distribution of the utilization category across the phylogeny and the proportion of total species examined (Fig. 1). When considering the use of PD to prioritize the conservation of threatened birds (Fig. 2), PD maximization generally outperforms extinction risk-based approaches alone at capturing currently utilized species. Our results indicate that maximizing overall PD can increase the chances of capturing species with certain uses to humans and is thus an effective strategy for the maintenance of future options. In addition, our results suggest that conserving threatened PD is essential to not only limit the loss of PD, and thus future options, but also to retain current benefits that would otherwise be neglected under either species extinction risk or functional prioritizations alone.

The efficiency of PD at capturing benefits varies depending on the number of species recorded and the distribution of the given benefit across the avian Tree of Life. PD performed strongest for the three benefits spread relatively widely across the avian phylogenetic tree: food, materials, and medicine (Fig. 1 and fig. S2). In contrast, the highly clumped distribution of species used as pets resulted in minimal gain from the maximization of PD (Fig. 1 and fig. S2). The phylogenetic clumping of utilization categories increased with their species richness (fig. S2), suggesting that PD provides particular utility at capturing rare benefits dispersed across the Tree of Life.

The large number of bird species used as pets (recorded for 73% of threatened birds and 35% of all birds in this study), which account for the majority (90%) of utilized bird species (*26*), combined with their highly clumped distribution on the bird phylogenetic tree and low ED, led to their considerable influence on the results when all benefits were combined. Although Passeriformes dominated utilized species, which is to be expected given the richness of the clade, the taxonomic distribution of utilized species varied by utilization category (fig. S2), with the second-highest utilized family differing between those used for food (Galliformes), materials (Pelecaniformes), medicine (Accipitriformes, following Strigiformes), and pets (Psittaciformes).

Despite the significant clumping and high prevalence of pet species (fig. S2), PD still provided significantly greater gains than random when all utilized species were combined at smaller sample sizes, where the pervasive presence of pet species was reduced compared with larger sample sizes (Fig. 1). Further information to disaggregate “pet” species into the constituent uses, e.g., whether species are kept as household pets or in zoological collections, would be valuable in decomposing the influence of pet species on the overall results. Highly evolutionarily distinct birds (top 10%) are more likely to be found in zoological collections than are other species (*27*).

Conserving threatened PD is increasingly recognized as an important endeavor, with trends in global PD being adopted as an indicator by The Intergovernmental Science-Policy Platform on Biodiversity and Ecosystem Services (IPBES) (*5*) and indicators for PD and the conservation status of EDGE species included in the Convention on Biological Diversity’s Kunming-Montreal Global Biodiversity Framework (*28*). Our results indicate that conserving threatened species based on PD captured more species with recorded benefits than by focusing on extinction risk alone (Fig. 2) and that species used by humans for medicine, materials, and food are more evolutionarily distinct than those that are not (table S2). Thus, whether through the EDGE metric—which combines ED with extinction risk (figs. S4 and S5) (*21*, *29*)—or through the maximization of PD irrespective of extinction risk categories (Fig. 2), prioritizing evolutionary history for conservation can provide an efficient means of maintaining both future options—through the maintenance of PD–and currently threatened benefits provided by birds globally.

There has been lively debate in academic literature discussing the value of PD as a conservation tool beyond a perceived surrogacy for other aspects of biodiversity, such as functional diversity (*30*–*33*). Our exploration of the relative performance of strategies to conserve threatened PD compared with sets of threatened species selected based on their FDist highlights that PD is comparably efficient at capturing benefits (Figs. 3 and 4, and fig. S6). Given the evidence that functionally distinct species are particularly targeted by consumptive practices such as hunting (*23*) and the pet trade (*25*), this result is unexpected as one can reasonably expect species sets selected based on FDist to effectively capture utilized species in our dataset.

Further, we found that, in addition to performing comparably to sets of species that prioritize functionally distinct birds, the utilized species captured by sets of species that prioritize evolutionarily distinct threatened birds shared low levels of overlap with those captured by sets of functionally distinct threatened birds (Fig. 5 and fig. S8). Various highly evolutionarily distinct, utilized, and threatened monotypic genera (e.g., maleo, *Macrocephalon maleo*; giant ibis, *Thaumatibis gigantea*; and subdesert mesite, *Monias benschi*) and families (e.g., the secretarybird, *Sagittarius serpentarius*; shoebill, *Balaeniceps rex*; and Kagu, *R. jubatus*)—many of which have important and unexpected utilitarian, cultural, and psychological value (*27*, *34*, *35*)—were overlooked when prioritizing by FDist alone. This was particularly pronounced when the number of threatened species to be conserved was low (e.g., 5%; Fig. 4). Conversely, many of the world’s most functionally distinctive birds would be overlooked if we focused solely on conserving evolutionary history. For example, the horned guan (*Oreophasis derbianus*), cape parrot (*Poicephalus robustus*), mariana crow (*Corvus kubaryi*), and several large fish eagles of the genus *Haliaeetus* would not be prioritized under an ED-based prioritization if we could conserve only 5% of threatened birds (Fig. 4).

Our findings suggest that the consideration of PD as simply a surrogate for other “desirable” aspects of biodiversity such as functional traits may require a rethink and that the unique value added by PD is in need of greater recognition. Prioritizing threatened species for conservation by their PD can effectively conserve option values, particularly when resources are restricted (i.e., smaller sample sizes in our study), and the potential benefits are rare and phylogenetically dispersed. Conversely, with unlimited resources to conserve a large proportion of threatened species, prioritizing by FDist would—at least for birds—be more efficient at capturing species associated with prevalent uses, particularly food and pets. However, given the restricted nature of conservation resources and the low overlap of utilized threatened species captured by evolutionary and functional metrics, conservation efforts should, where possible, prioritize both evolutionarily and functionally distinct species to effectively safeguard their associated benefits to humans (Fig. 5).

However, whereas phylogenetic trees are increasingly available for large parts of the Tree of Life (*36*–*40*), comprehensive functional trait datasets are not yet available for the vast majority of species (*41*, *42*). Thus, the gap between phylogenetic and functional data availability therefore needs to be bridged to facilitate the use of functional data in conservation prioritizations as readily as can be achieved with PD. Here, we used FDist scores for birds derived from a limited number of semiquantitative and categorical traits, with body size as the only continuous measure (*43*, *44*). It is common for studies of functional diversity to rely on limited amounts of primarily categorical traits (*42*, *45*) and on traits that are often phylogenetically clumped (*46*). However, continuous trait data are increasingly available for the world’s birds (*47*) and other vertebrates (*48*, *49*). We hope that our findings will inspire further explorations into how the use of continuous trait datasets influences how utilized species (or any species of interest) are represented in trait space and explore how the unsustainable use of species will erode functional diversity at local and global scales.

It is reasonable to assert that known benefits today, such as the utilization data used for our study, were at one point in human history unknown future options. Had we taken a time machine to conduct this research at a remote past point in time when the current utility of bird species was not yet realized (e.g., paleolithic societies) and selected the sets of species to maximize PD with no knowledge of their future benefits, we would have performed better at capturing species with future benefits to humans—particularly benefits that are relatively rare and phylogenetically dispersed—than when selecting species at random. Our findings thus complement earlier work on plants at both a local (*13*) and global scale (*14*) by providing empirical evidence for the link between evolutionary history and the option value of biodiversity.

It is important to recognize that, for many species, their value to people is now also a serious threat. The unsustainable use of species has contributed to some bird species becoming extinct (e.g., the passenger pigeon, *Ectopistes migratorius*) (*50*), and today is one of the largest drivers of species population declines (*51*) and extinction risk (*18*, *26*). When combined with other threats, previously sustainable practices have the potential to drive species to extinction (*34*, *52*). However, there is also good evidence that, when well-managed, the use of wild species can be sustainable and contribute positively to their conservation (*53*). Our findings highlight that, while it is important to maintain diversity to ensure current and future benefits can be realized, the scale of the biodiversity crisis necessitates the sustainable use of species to guarantee their survival (*3*, *6*). The species highlighted here (Figs. 4 and 5, and data S1) are some of the most distinctive and threatened species on Earth and the conservation and sustainable use of which are imperative to avert large losses of irreplaceable phylogenetic and functional diversity.

Our work was limited to species for which some form of use or trade had been recorded by an IUCN Red List assessor as part of the assessment process and thus is not an exhaustive examination of the uses and benefits provided by birds to humanity. It is likely that many recorded consumptive uses of birds by humans have been overlooked, such as the use of colorful feathers for various ornamental and cultural reasons (*54*). While this work focuses only on direct benefits derived from birds, future work should also explore the link between evolutionary history and the wide variety of indirect benefits we receive from biodiversity, including those tied to ecosystem functioning, cultural values, and psychological well-being at the local and global scales (*1*, *3*, *8*, *55*). In addition, more research is needed to explore how the distribution of benefits across the phylogeny affects the utility of PD for capturing beneficial species. Last, birds represent only a small part of the role biodiversity plays in humanity’s relationship with nature, and further research across the Tree of Life is required.

Our study highlights the importance of conserving PD to ensure we maintain a diversity of options for the future. We must strive to retain variety across multiple dimensions of biodiversity if we are to be truly ambitious about halting the loss of biodiversity for future generations and the known and yet-to-be-discovered benefits it bestows.

## MATERIALS AND METHODS

### Experimental design

#### Identifying species of importance

We restricted the taxonomic scope of our analyses to birds for two reasons: (i) their almost complete data coverage (>99% of species) and adequate recording of use and trade data on the IUCN Red List (*26*) and (ii) their high phylogenetic coverage (>85% of species included in a published phylogenetic tree) (*56*). Although there is now somewhat comparable phylogenetic and IUCN Red List coverage for other vertebrate classes, particularly mammals and amphibians, the current IUCN Red List mammal use and trade data are considered inadequate for these types of analyses (*26*).

For our analyses, we extracted and categorized the benefits to humans provided by bird species using the IUCN Red List use and trade classification scheme (www.iucnredlist.org/resources/general-use-trade-classification-scheme), which identifies 16 specific “consumptive uses” of species (Table 1). For our analyses, we considered only those consumptive uses that could be directly reconciled with one or more of the 18 broader categories of Nature’s Contributions to People (NCPs) identified by IPBES (IPBES 2019). We then grouped the IUCN categories of uses of species (hereafter “IUCN uses”) based on these broader IPBES NCP category associations, and some uses were mapped to more than one NCP (Table 1).

**Table 1.** The 16 specific IUCN uses defined by the IUCN Red List, their purpose—determined from the description/definition given by the IUCN Red List, the broader IPBES NCP categories to which each use was linked, and the number of extant species and threatened (CR, EN, or VU on the Red List) species with phylogenetic information included in this study. “-” indicates zero species were listed under the given use by the IUCN Red List. Total species refer to unique species, whereas a species may occur in multiple use categories; hence, the total value is less than the sum of the rows.

IUCN use	IPBES NCP category	Species	Threatened spp.
1. Food (human)	Food	1402	346
2. Food (animal)	Food	-	-
3. Medicine (human and veterinary)	Medicinal	57	15
4. Poisons	Medicinal	-	-
5. Manufacturing chemicals	Medicinal	-	-
6. Other chemicals (e.g., perfume)	Materials	-	-
7. Fuels	Energy	12	3
8. Fiber	Materials	-	-
9. Construction or structural	Materials	-	-
10. Wearing apparel, accessories	Materials	-	-
11. Other household goods	Materials	47	21
12. Handicrafts, jewelry, etc.	Materials	66	17
13. Pets/display animals, horticulture	Pets (NCPs 13, 15, 16)	3855	441
14. Research	Medicinal	1	-
15. Sport hunting/specimen collecting	-	402*	62*
16. Establishing ex situ production	-	1*	-*
**Total species**		**4291 (4331** **^†^** **)**	**604 (608** **^†^** **)**

*Use categories that were excluded from the main analyses due to lack of sufficient detail to link the use to a broader utilization category.

†The total of unique species when all uses are included.

We identified utilized bird species that could reliably be grouped into four broad utilization categories: fuel/energy, food, materials (handicrafts, jewelry and other household goods), and medicine (Table 1). As there was insufficient information to disaggregate 99% (4482 spp.) of pet records into their reason for use (e.g., personal pet or Zoo collection), we also included pets and display animals combined as a single group. As there were just 12 bird species utilized for energy (just three of which are threatened; use 7, Table 1), we did not analyze species used for energy independently from the overall dataset of utilized species.

For our analyses, we extracted a sample of 100 phylogenetic trees from the distribution of 10,000 species-level bird trees of Jetz *et al.* (*56*) and matched the species to the 2019 BirdLife International taxonomy for birds (*57*), which, as the Bird Red List Authority, establishes the Red List taxonomy for birds on the Red List. Where species could not be matched from the trees to the taxonomy, they were removed from the phylogenetic trees, resulting in a set of 100 phylogenetic trees comprising 9645 species. We conducted all the analyses described below and averaged results across the 100 phylogenetic trees to account for phylogenetic uncertainty (*14*, *58*). For our main analyses, we restricted our set of utilized species to those species recorded in the food, materials, medicine, and pet categories, which could also be matched to species in our updated trees (4291 spp.; Table 1). However, we also ran additional analyses for species linked to IUCN use 15, “sport hunting/specimen collecting,” which we could not reconcile with any broader category due to insufficient information, and reran our analyses of all utilized species with all species with recorded uses included, irrespective of direct link to a broader utilization category, to explore the impacts of including all utilized species on the results (data S1).

#### Avian evolutionary history and utilized species

We calculated the number of species in each bird order associated with each utilization category and compared this with the null expectation of taxonomic distribution of utilized records if their distribution was at random across all birds. To do this, we selected 999 sets of bird species of equal number to those observed for each utilization category at random from all bird species and calculated the number of species from each bird order. We considered an order to be significantly overrepresented or underrepresented in a utilization category when the observed number fell within the highest 97.5% or lowest 2.5% of simulated values, respectively.

We used the greedy algorithm (*32*, *59*) to find sets of species to maximize PD (i.e., finding the longest path across a tree to connect all species in a given set for size *S* to maximize branch lengths captured) at sample sizes *S* of 2.5, 5, 10, 25, and 50% of the total number of species in the tree. As there are multiple sets of species that can maximize PD for a given sample size, we generated 10 PD-maximizing species sets for each of the 100 phylogenies, resulting in 1000 species sets for each sample size [following Molina-Venegas *et al.* (*14*)]. We then generated a null distribution of 1000 random species sets for each sample size for comparison with our PD-maximizing sets. For each sample size, we then calculated how many species recorded for each utilization category were captured by PD-maximizing sets and random sets of species, respectively (both for each utilization category separately and all combined). To generate comparable results across sample size *S*, we calculated standardized effect sizes (SESs) for each sample size following Molina-Venegas *et al.* (*14*) using

$$\mathrm{SES}=\frac{M_{\mathrm{PD}}-M_{\mathrm{null}}}{{\mathrm{SD}}_{\mathrm{null}}}$$

where SES is the SES score for a given PD approach and sample size, *M*_PD_ is the observed mean value of the variable when species selection is phylogenetically informed, *M*_null_ is the mean of the null distribution, and SD_null_ is the standard deviation of the null distribution.

To assess the phylogenetic dispersion of species associated with each utilization category, we calculated the PD captured by species used for each category and all combined for each of the 100 phylogenetic trees. We then generated a null distribution of PD for each tree by shuffling species names across the tree tips and recalculating PD 1000 times and calculated an SES score as above for each utilization category. We took the mean SES score for each utilization category versus null distribution, following Molina-Venegas *et al.* (*14*), where high SES scores indicate greater dispersion of utilized species, and lower SES scores indicate phylogenetic clumping. We also calculated median ED scores (*21*) for all birds from the 100 phylogenetic trees used for the PD analyses. We then explored whether species associated with each utilization category differed in their ED scores.

#### Prioritizing threatened avian evolutionary history to safeguard utilized species

Extinction risk must also be considered when identifying sets of species to conserve, as PD-maximizing sets may not necessarily represent the optimal sets to avert the loss of threatened PD. For example, a set of 100 bird species that are selected to maximize PD could, in theory, all be listed as least concern (LC) by the IUCN Red List and in no immediate danger of becoming extinct. We therefore incorporated extinction risk in two ways to explore the performance of conservation strategies that aim to avert the loss of PD at capturing threatened and utilized species.

First, we considered all bird species listed as LC or near threatened (NT) by the IUCN Red List to be currently at low risk of extinction and all species in threatened Red List categories—VU, EN, CR, and extinct in the wild (EW)—to be “threatened.” We considered “low-risk” species as secured from extinction and then again applied a greedy algorithm to identify sets of threatened species that, if conserved, would maximize the gain in PD in addition to that secured by the low-risk species. We generated 1000 sets of PD-maximizing threatened species at sample sizes of 2.5, 5, 10, 25, and 50% of total threatened species in our phylogeny (1491 spp.).

Second, we again considered LC and NT species to be low risk and selected sets of threatened species to “conserve” using the original “EDGE” approach. The EDGE approach prioritizes species based on the combined magnitude of their ED and extinction risk or GE (*21*). High-ranking EDGE species are threatened species that are likely to be responsible for preserving large amounts of unique PD into the future, and high ED plant species have been shown to capture multiple benefits (*16*). Averting the extinction of high-ranking EDGE species can therefore avert the greatest losses of PD. We selected sets of threatened bird species based on their EDGE ranks—selecting the highest-ranking species first—again at sample sizes of 2.5, 5, 10, 25, and 50% of total threatened species. We generated 100 EDGE scores for the threatened species from a sample of 100 phylogenies from the distribution of Jetz *et al.* (*56*).

To assess the performance of the PD-maximizing and EDGE strategies for capturing threatened species recorded in utilization categories, we generated distributions of species captured at the same sample sizes for comparison, under two alternative sampling strategies. First, we generated a distribution of 1000 sets of threatened species for each sample size, whereby the probability of a species being selected for conservation was weighted by their IUCN Red List category (i.e., species in more severe extinction risk categories had a higher probability of being selected, with weightings following the IUCN Red List Index weightings of categories: VU = 2, EN = 3, CR = 4, EW = 5), hereafter referred to as the “RL-weighted” strategy, under the assumption that species with higher risk of extinction are more likely to receive conservation action (*60*).

Second, we implemented a sampling strategy where subsets of threatened species are sampled at random but conditioned to meet the proportion of threat categories sampled by each PD-maximizing subset of threatened species. For example, if a set of PD-maximizing species was composed of 20% CR, 50% EN, and 30% VU species, then we generated a null set of species for comparison that selected species at random from each Red List category to produce 20% CR, 50% EN, and 30% VU species. We repeated this for each sample size across the 1000 iterations and hereafter refer to the approach as the “RL-controlled” strategy. This strategy controls for any potential relationship between phylogeny and extinction risk, allowing the comparison of PD-maximizing sets against sets of threatened species in general. We used the same approach as before to determine the relative change in utilized species captured and SES scores for each sample size.

We created a third null distribution of species, derived from FDist—a measure of the isolation of species in functional space—for comparison against the PD maximization approach, under the assumption that species with “unusual” traits are more likely to be targeted by humans for various uses (*24*, *25*). To achieve this, we matched the species in our dataset to the FDist scores from Pollock *et al.* (*44*), who used traits on diet, foraging strategy, activity patterns, and body size to generate FDist measures for all bird species. We used these FDist scores to weight the probability of a species being selected for conservation. We again generated SES scores between the PD maximization and FDist-weighted approaches.

#### Conserving distinctive and utilized species

Last, we explored the performance of prioritizing the most evolutionarily and functionally distinct species at capturing utilized threatened species by picking species in decreasing order of ED and FDist individually. We estimated median ED scores for all birds from the 100 phylogenetic trees used for the PD analyses. We quantified the accumulated benefits and extent of overlap between the utilized species captured in each approach for the same sample sizes as PD-based test above. We then explored whether species used for each category, and all categories combined, were more “distinctive” than species with no recorded association to each utilization category.

### Statistical analyses

To determine statistical significance from the SES results, we followed Molina-Venegas *et al.* (*14*) by determining that, for a 5% nominal α, SES scores above +1.96 and below −1.96 indicate that the observed results (e.g., number of utilized species captured) were significantly larger or smaller than random expectation, respectively. To determine whether the ED scores differed between species in each utilization category, we used Kruskal-Wallis with Dunn’s test for pairwise comparisons. We compared ED and FDist scores of utilized species versus non-utilized species using Welch’s *t* tests. We tested for a relationship between ED and FDist of all birds, and threatened birds only, using Pearson’s correlations. All statistical analyses were conducted in R (*61*) using the packages Caper (*62*) and dunn.test (*63*), as well as the “greedyPD” function developed by Mazel *et al.* (*32*).
