# Supplementary material for: Conserving avian evolutionary history can effectively safeguard future benefits for people
Source: Sci Adv. 2023 Sep 20;9(38):eadh4686. doi: 10.1126/sciadv.adh4686 (PMC10511189; doi:10.1126/sciadv.adh4686)
Supplement: Supplementary file 1 — Figs. S1 to S8 Tables S1 to S3 Legend for data S1 [file sciadv.adh4686_sm.pdf]

Science Advances
AAAS

Supplementary Materials for

Conserving avian evolutionary history can effectively safeguard future benefits for people

Rikki Gumbs *et al.*

Corresponding author: Rikki Gumbs, rikki.gumbs@zsl.org

Sci. Adv. **9**, eadh4686 (2023)
DOI: 10.1126/sciadv.adh4686

The PDF file includes:

Figs. S1 to S8
Tables S1 to S3
Legend for data S1

Other Supplementary Material for this manuscript includes the following:

Data S1

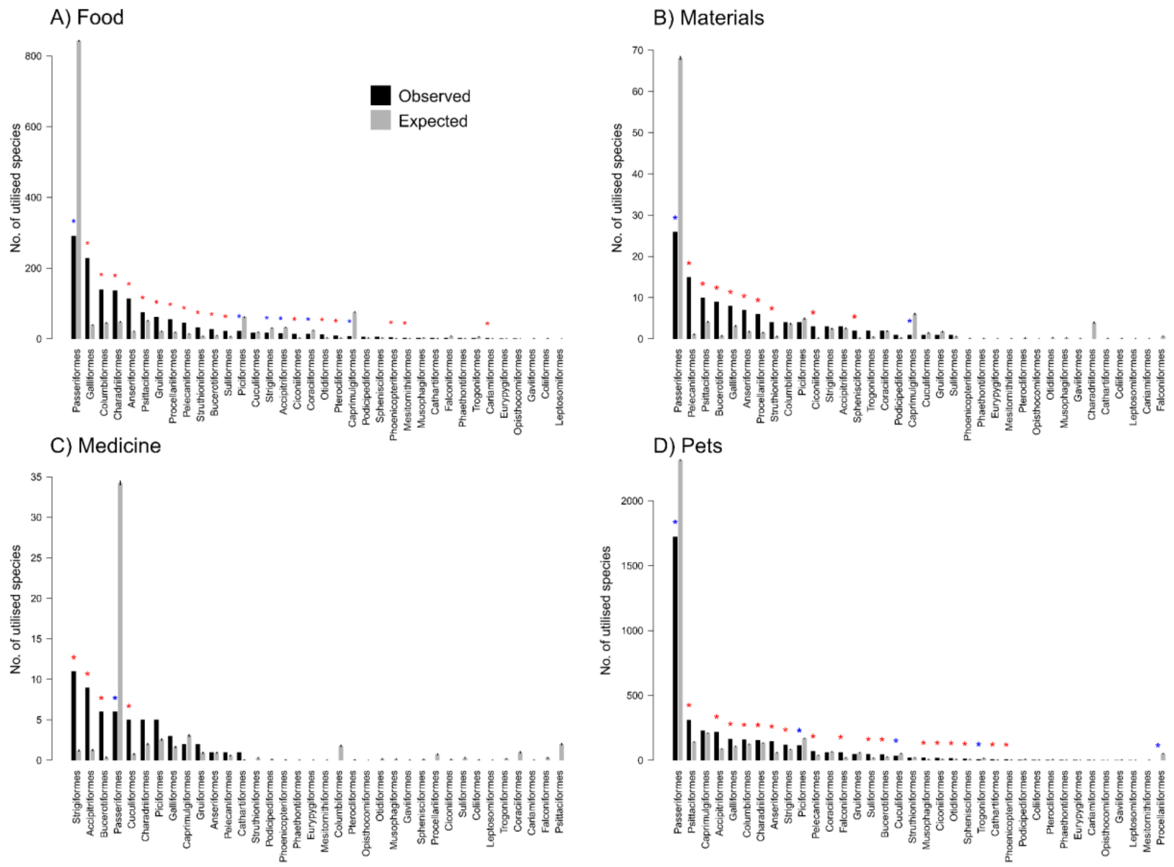

Fig. S1. Distribution of recorded uses of birds across orders. For the four main utilisation categories: (A) food; (B) materials; (C) medicine; and (D) pets, the number of observed utilised species in each order (black bars) versus the mean expected number of utilised species in each order (grey bars with 95% confidence interval bars). The expected number is derived from 999 calculations where sets of species equal in size to those observed to be utilised for each category were drawn at random from all bird species. Red asterisks denote orders that are significantly overrepresented compared to the random expectation, whereby the observed value is in the top 2.5% of all values from the simulated set. Conversely, blue asterisks denote orders that are significantly underrepresented compared to the random expectation, whereby the observed value is in the lowest 2.5% of all values from the simulated set.

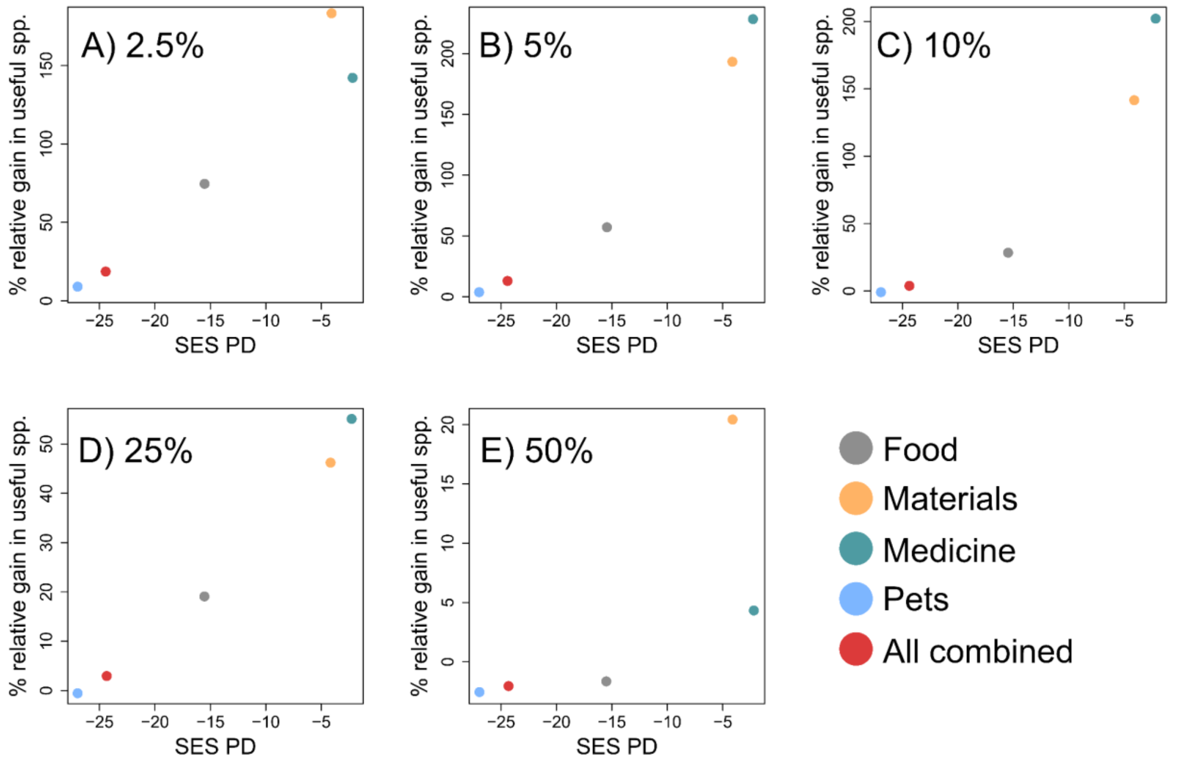

Fig. S2. The relationship between phylogenetic dispersion and relative gains in utilised species across the phylogenetic tree. PD SES scores provide a measure of dispersion of utilised species across the tree relative to random, and were averaged across 100 phylogenetic trees, and compared to the observed relative gain in utilised species at sample sizes of (a) 2.5%, (b) 5%, (c) 10%, (d) 25%, and (e) 50% of species, for each utilisation category and all categories combined.

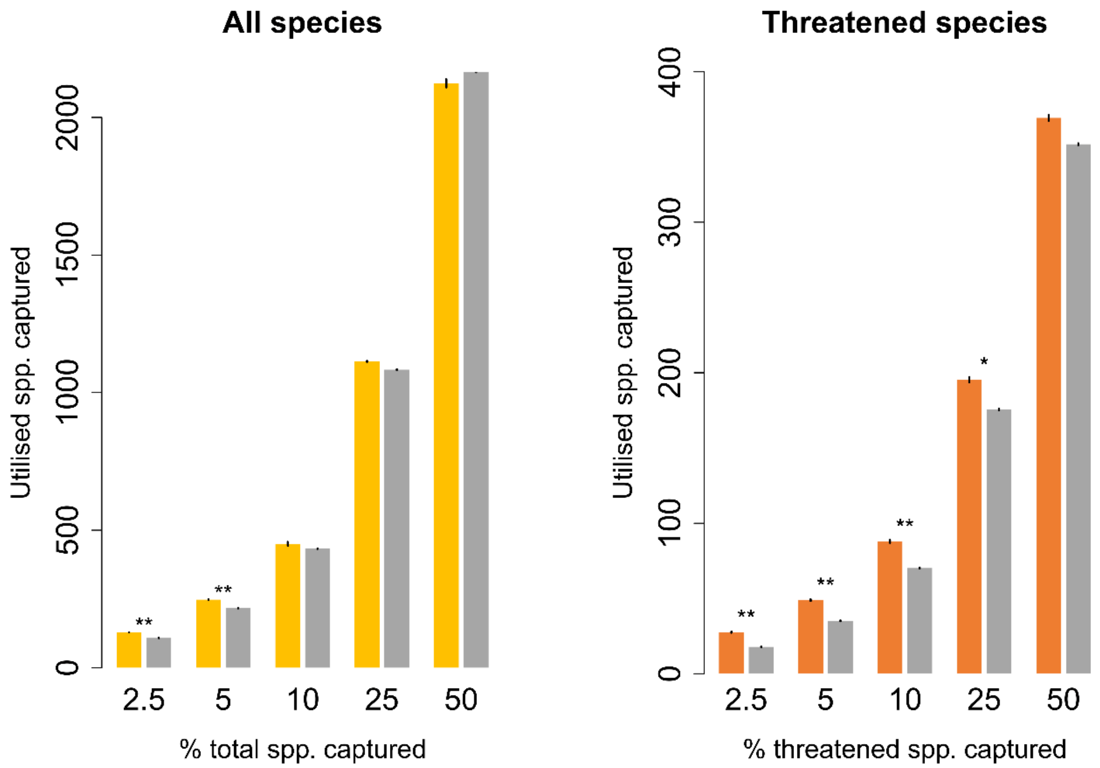

Fig. S3. Results from Fig. 2 and Fig. 3 when all utilsation records included. The number of threatened and utilised species captured by maximising PD for all species (left) and threatened species (right), compared with the numbers saved when species are selected at random (left; grey bars) and when weighted by Red List category (right; grey bars). Significance based on SES scores, for alpha of 5% (*), 1% (**) and 0.01% (***). Error bars signify 95% confidence interval around the mean for 100 values.

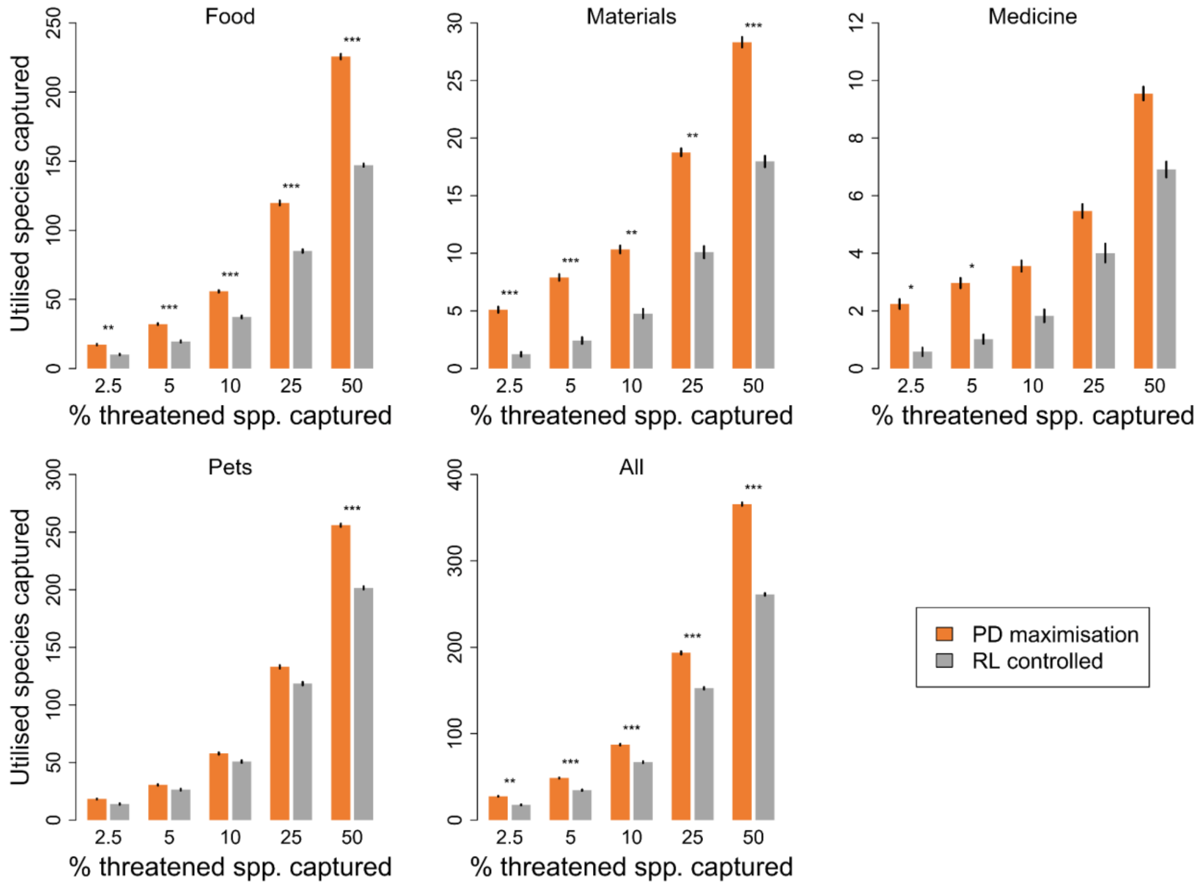

Fig. S4. The number of threatened and utilised species captured by maximising the conservation of threatened PD, compared with the numbers saved when Red List category is controlled for (i.e. equal to the distribution of Red List categories in the PDmax data) in the random selection of species for the null distribution. Significance based on SES scores, for alpha of 5% (*), 1% (**) and 0.01% (***). Error bars signify 95% confidence interval around the mean for 100 values.

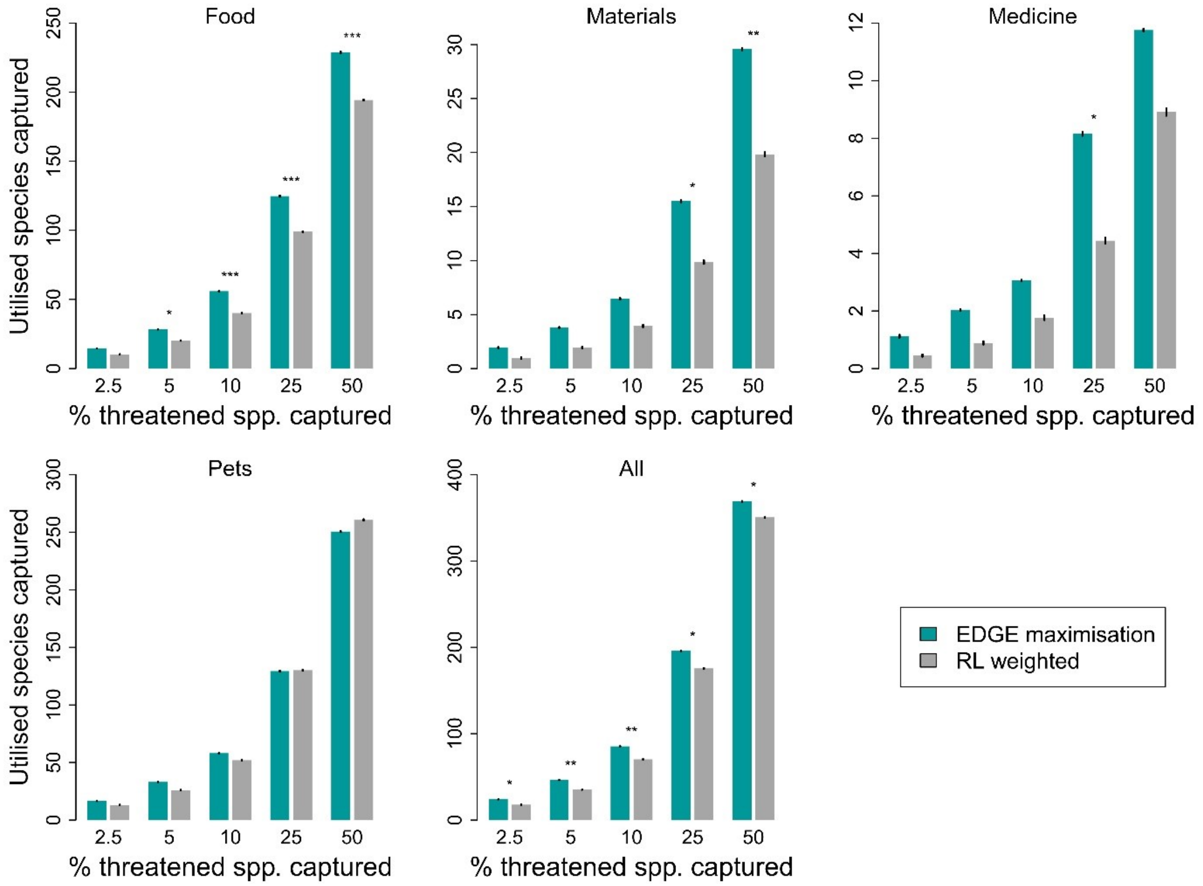

Fig. S5. The number of threatened and utilised species captured by selecting species based on their EDGE rankings, compared with the numbers saved when species are selected based on their IUCN Red List category. Significance based on SES scores, for alpha of 5% (*), 1% (**) and 0.01% (***). Error bars signify 95% confidence interval around the mean for 100 values.

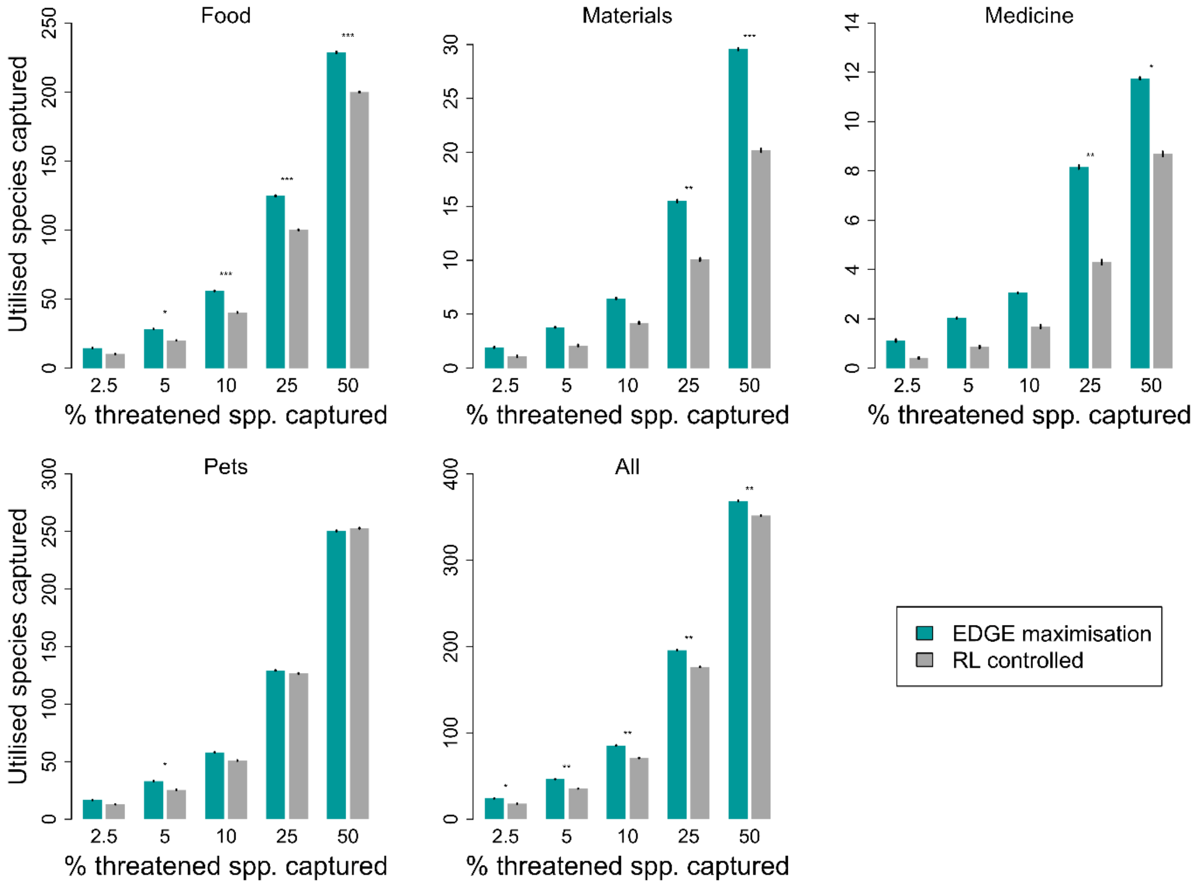

Fig. S6. The number of threatened and utilised species captured by selecting species based on their EDGE rankings, compared with the numbers saved when Red List category is controlled for (i.e. equal to the distribution of Red List categories in each EDGE set) in the random selection of species for the null distribution. Significance based on SES scores, for alpha of 5% (*), 1% (**) and 0.01% (***). Error bars signify 95% confidence interval around the mean for 100 values.

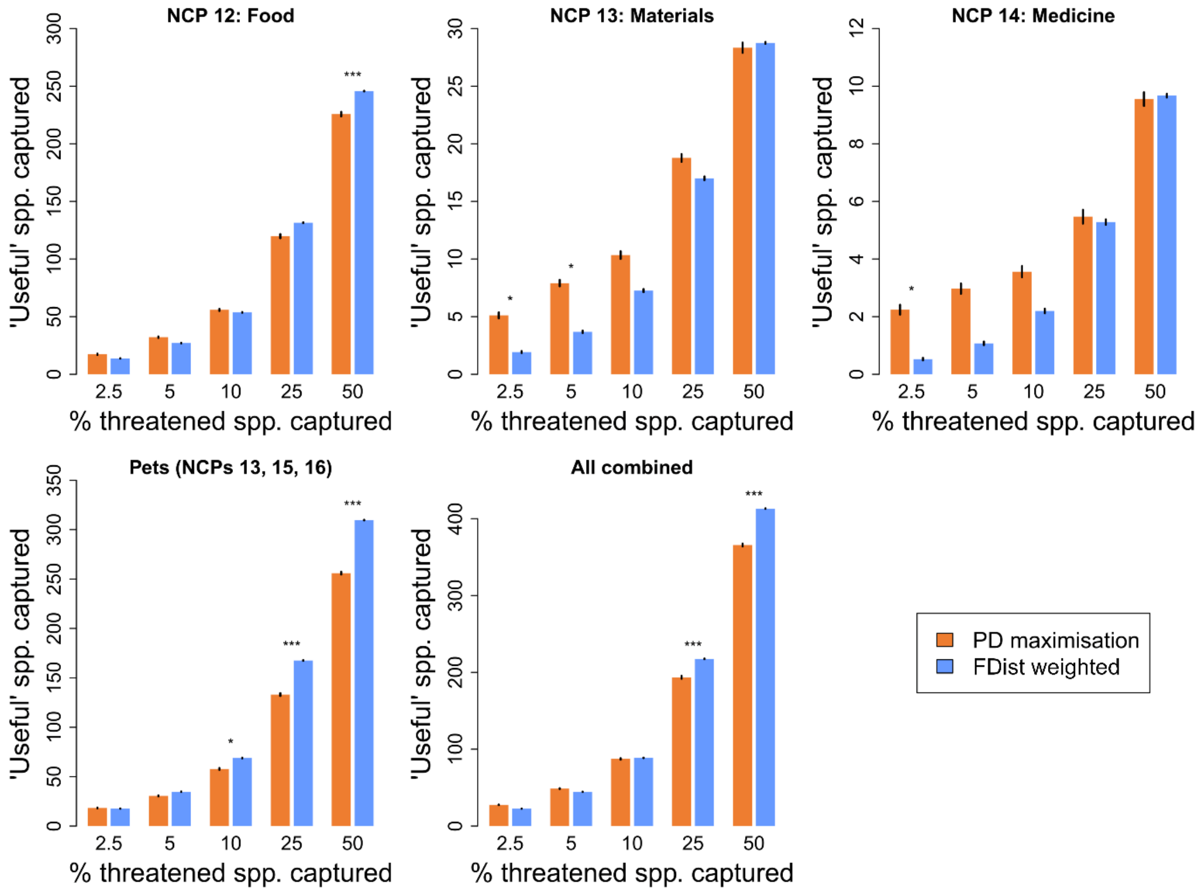

Fig. S7. The number of threatened and utilised species captured by selecting species to maximise the threatened PD captured, compared with the numbers saved when species are selected based on their functional distinctiveness (FDist) rankings. Significance based on SES scores, for alpha of 5% (*), 1% (**) and 0.01% (***). Error bars signify 95% confidence interval around the mean for 100 values.

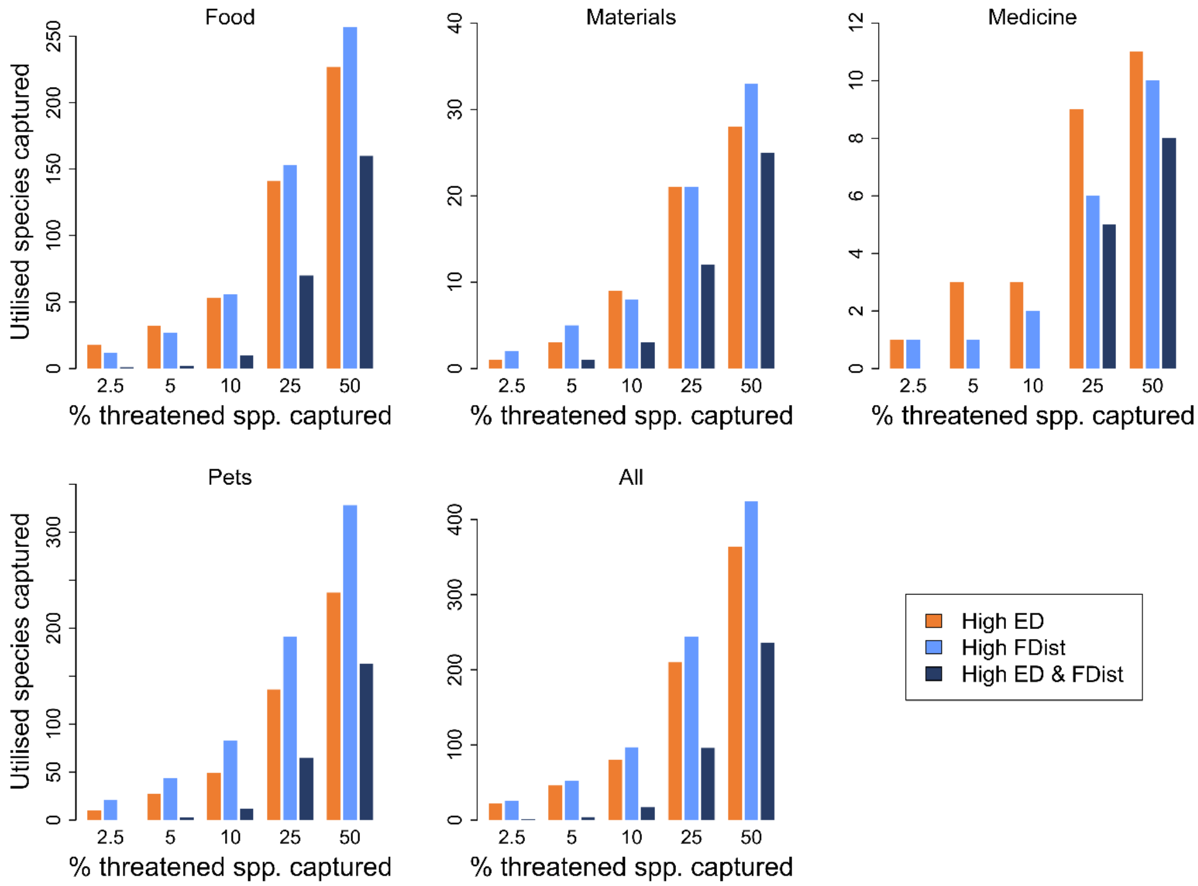

Fig. S8. The number of threatened and utilized species for each utilization category, and all combined, captured when species are selected based on their evolutionary distinctiveness (ED) or functional distinctiveness (FDist) scores, and the overlap of species captured by both distinctiveness metrics. Threatened species were ranked by their ED or FDist score and the number of utilised species captured at each sample size was determined.

Table S1. Differences in evolutionary distinctiveness (ED) scores between utilisation categories. Results from Dunn's test for post-hoc pairwise comparison between groups in a Kruskal-Wallis, comparing the ED scores of species recorded as utilised for food, materials, medicine and pets.

Comparison	Z	Adjusted p value
Materials-Food	-3.323	0.027
Medicine-Food	-1.196	0.695
Pets-Food	6.453	3.28×10^{-10}
Medicine-Materials	1.026	0.914
Pets-Materials	5.505	1.11×10^{-7}
Pets-Medicine	2.733	0.018

Table S2. Evolutionary distinctiveness of utilised versus non-utilised species. Results from Welch's t-Test for comparison of ED for utilised versus non-utilised species across all utilisation categories, and all combined, for all species (9,645 spp.) and for threatened species only (1,491 spp.). Bold p values = significant difference following Bonferroni correction.

Use	Species set	Mean ED (utilised species)	Mean ED (non-utilised species)	t	d.f.	p
Food	All	9.34	7.61	8.87	1912.78	**1.56×10^{-18}**
Materials	All	11.32	7.82	5.27	124.82	**5.72×10^{-7}**
Medicine	All	10.85	7.84	2.39	63.25	**1.95×10^{-3}**
Pets	All	7.96	7.79	1.38	8867.32	0.164
All combined	All	8.18	7.6	5.04	9548.72	**4.53×10^{-7}**
Food	Threatened	9.82	7.37	5.35	504.36	**1.29×10^{-7}**
Materials	Threatened	12.23	7.9	3.28	37.43	**2.19×10^{-3}**
Medicine	Threatened	14.14	7.96	1.49	14.05	0.156
Pets	Threatened	8.45	7.81	1.57	758.59	0.115
All combined	Threatened	8.98	7.19	4.81	1084.68	**1.64×10^{-6}**

Table S3. Functional distinctiveness (FDist) of utilised versus non-utilised species. Results from Welch's t-Test for comparison of FDist for utilised versus non-utilised species across all utilisation categories, and all combined, for all species (9,645 spp.) and for threatened species only (1,491 spp.). Bold p values = significant difference following Bonferroni correction.

Use	Species set	Mean FDist (utilised species)	Mean FDist (non-utilised species)	t	d.f.	p
Food	All	0.039	0.027	14.76	1912.78	**2.41×10^{-46}**
Materials	All	0.05	0.028	7.48	124.82	**3.09×10^{-11}**
Medicine	All	0.041	0.028	3.7	63.25	**5.21×10^{-4}**
Pets	All	0.037	0.023	24.37	8867.32	**1.88×10^{-125}**
All combined	All	0.037	0.022	25.5	9548.72	**2.30×10^{-137}**
Food	Threatened	0.039	0.026	7.33	504.36	**7.52×10^{-13}**
Materials	Threatened	0.05	0.029	4.09	37.43	**2.27×10^{-4}**
Medicine	Threatened	0.038	0.029	1.17	14.05	0.261
Pets	Threatened	0.04	0.024	10.05	758.59	**2.06×10^{-22}**
All combined	Threatened	0.037	0.023	10.16	1084.68	**2.57×10^{-23}**

Data S1. (separate file)
All underlying data for the plots from the main text and supplementary materials (R Data file).
